# Supplementary material for: Evidence for a Spoken Word Lexicon in the Auditory Ventral Stream
Source: Neurobiol Lang (Camb). 2023 Jul 20;4(3):420–34. doi: 10.1162/nol_a_00108 (PMC10426387; doi:10.1162/nol_a_00108)
Supplement: Supplementary file 1 [file nol-4-3-420-s001.pdf]

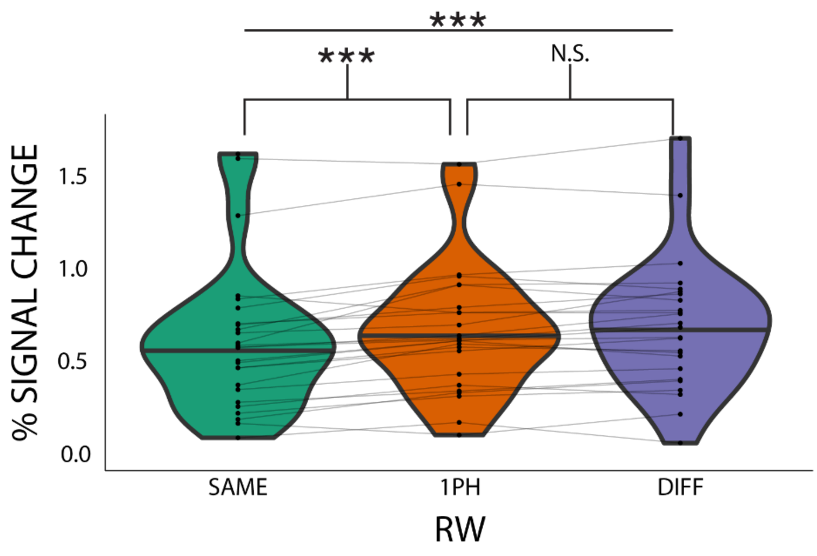

Figure S1. Evidence for auditory lexical representations in the auditory word form area. Adaptation profile for auditory real words (RWs) using the full data set (*n* = 26), including those (*n* = 2) without corresponding untrained pseudoword scans. RWs show tight tuning to individual RWs consistent with an auditory lexicon. Horizontal black line in violin plots indicates the median. ***, **, *, and N.S. mark *p* <0.001, < 0.01, < 0.05, and not significant (>0.1), all Bonferroni-corrected for multiple comparisons.

Table S1. Individual subject AWFA ROIs

Subject	X-coordinate	Y-coordinate	Z-coordinate
sub-MR1076	–62	–16	2
sub-MR1106	–62	–16	4
sub-MR1215	–62	–16	4
sub-MR1216	–62	–12	0
sub-MR1217	–64	–20	4

sub-MR1220	–58	–16	0
sub-MR1221	–64	–14	4
sub-MR1222	–64	–10	0
sub-MR1233	–62	–12	8
sub-MR1234	–62	–12	6
sub-MR1240	–66	–16	6
sub-MR1242	–62	–16	0
sub-MR1252	–60	–10	2
sub-MR1253	–60	–10	0
sub-MR1258	–58	–12	0
sub-MR1272	–62	–18	2
sub-MR1273	–58	–18	6
sub-MR1277	–64	–18	2
sub-MR1278	–66	–22	4
sub-MR1282	–60	–12	–2
sub-MR1283	–62	–18	4
sub-MR1284	–60	–16	4
sub-MR1286	–64	–12	–2
sub-MR1287	–60	–16	6
sub-MR1289	–66	–20	2
sub-MR1294	–62	–10	2

Table S2. Significant clusters of connectivity with the AWFA

X-coordinate	Y-coordinate	Z-coordinate	Size (mm^3)	*p* FDR
64	–20	0	5239	0
–64	–28	8	5217	0
–6	12	48	970	0
50	–2	50	934	0
–12	26	58	800	0
–46	12	26	799	0
–40	–76	36	737	0
–34	24	8	429	0
18	–24	–4	351	0
–26	–62	–24	272	0
46	–68	28	258	0
12	–70	–18	256	0
34	30	54	242	0
–4	–92	4	203	0
40	–80	8	134	0
–62	–24	–14	100	0
–44	–46	–14	100	0
8	–60	68	67	0.000007

14	–92	0	63	0.000012
38	–52	48	60	0.000018
–30	–52	40	57	0.000026
6	54	16	52	0.000055
–10	–52	28	48	0.000101
–12	–66	8	45	0.00016
–6	–32	28	43	0.000206
8	8	6	43	0.000206
20	8	0	41	0.00028
24	–52	6	39	0.000383
–30	–2	56	38	0.000426
46	–58	–26	38	0.000426
26	–38	–16	32	0.001193
–6	–18	8	32	0.001193
–24	–40	–8	27	0.003037
–44	4	–36	23	0.006664
–10	–66	–16	22	0.007996
56	–24	32	21	0.009372
50	–30	56	21	0.009372
–8	58	12	18	0.017283
–6	50	10	18	0.017283
34	–52	–14	17	0.02019
–32	16	–16	17	0.02019

8	–28	30	17	0.02019
–2	–4	20	16	0.023298
–40	–48	42	16	0.023298
6	–14	30	16	0.023298
8	–34	–28	16	0.023298
–48	–36	50	15	0.02896
–32	–36	–18	14	0.034761
18	–82	12	14	0.034761
–6	–70	–26	14	0.034761
